# Mink SLAM V-Region V74I Substitutions Contribute to the Formation of Syncytia Induced by Canine Distemper Virus

**DOI:** 10.3389/fvets.2020.570283

**Published:** 2021-01-21

**Authors:** Yawen Wang, Jie Chen, Bo Hu, Chengyan Gong, Ning Shi, Mengjia Liu, Xijun Yan, Xue Bai, Jianjun Zhao

**Affiliations:** ^1^Key Laboratory of Special Animal Epidemic Disease, Ministry of Agriculture, Institute of Special Animal and Plant Sciences, Chinese Academy of Agricultural Sciences (CAAS), Changchun, China; ^2^Department of Microbiology and Immunology, College of Veterinary Medicine, China Agricultural University, Beijing, China; ^3^Dongying Customs District, People's Republic of China, Dongying, China; ^4^College of Animal Science and Veterinary Medicine, Heilongjiang Bayi Agricultural University, Daqing, China

**Keywords:** canine distemper virus, signaling lymphocyte activation molecule, hemagglutinin protein, syncytia, variable region

## Abstract

The Signal lymphatic activation molecule (SLAM, also known as CD150) as the cellular receptor of canine distemper virus (CDV) plays an important role in the virus-host interaction. However, it is still unknown whether amino acid differences in the SLAM variable (V) region affect the formation of syncytia. Here, using raccoon dog SLAM (rSLAM) and mink SLAM (mSLAM), we performed SLAM-V homologous modeling, site-directed mutagenesis, and surface expression analysis, as well as a cell fusion assay, to study the interaction between SLAM and CDV. More specifically, our investigation focused on two amino acid residues (74 and 129) of SLAM, previously predicted to play a relevant role in receptor-ligand interaction. Our results indicated that only residues at position 60, 74, and 129 were different between rSLAM and mSLAM among the 29 amino acids that might interact with CDV H, and residues 74 and 129 were located in the interface region interacting with CDV H. The amino acid substitution at the positions of 74 have a significant effect on the expression of mSLAM. The SLAM-V74I mutation in mink significantly improved the cell fusion efficiency of CDV. In contrast, the SLAM-I74V mutation in the raccoon dog significantly decreased cell fusion efficiency. We conclude that residue 74 of SLAM plays an important role during the the formation of syncytia. Only when implementing CDV infection analysis, the rSLAM-Q129R can significantly decreased the mean number of syncytia, but the mSLAM-R129Q can't. Additionally, residue 60 show variability between rSLAM and mSLAM. We believe that our study makes a significant contribution to the literature because we provide molecular data, partially accounting for the differences in host membrane and virus interaction laying the foundation for further molecular work.

## Introduction

Canine distemper (CD) is a highly contagious disease with high mortality caused by the canine distemper virus (CDV), which infects a wide variety of carnivores, including domestic dogs and an assortment of wildlife hosts (1, 2). The CDV host range is continuously expanding as the ecological environment changes and the virus evolves (3–6). SLAM identified as the cellular receptors of CDV (7–11) play critical roles in the cross-species infection based on their interaction with the CDV hemagglutinin (H) protein (12–16). The SLAM receptor cooperates with tetrameric H proteins to trigger cell fusion for viral entry in the presence of trimeric fusion (F) proteins (17–19).

The affinity of morbillivirus to different species may depend on differences in key amino acids in the SLAM protein. Shimizu et al. (20) identified 32 amino acids of the SLAM protein that are implicated in the recognition of the measles virus H protein in aquatic animals, with bottlenose and striped dolphins having substitutions at five positions (E68G, I74V, R90H, V126I, and Q130H) compared with baleen whales. Three residues (at positions 68, 90, and 130) were found to alter electric charges, with the potential to modify virus affinity for host cells (20). In 2013, Ohishi et al. (21) found that substitution of four residues with those having altered charge at positions 72, 76, 82, and 129 in the dog SLAM interface region compared with that of the domestic cat appeared to contribute to differences in affinity for CDV. These findings demonstrate that there are species-specific variations in the amino acid residues on the SLAM surface, raising the possibility that some might be key residues required for binding affinity and sensitivity to the virus.

In our previous studies, the models of raccoon dog and mink infected with LN(10)1 srain have been established (22). To further study the effect of receptor on cell fusion efficiency, LN(10)1 was selected as the target strain in this study based on this pathogenic models of it infecting raccoon dogs and minks. The SLAM-dependent cell–cell fusion plays key roles in virus tropism and virulence *in vivo* as well as cytopathogenicity *in vitro* (23, 24). The cell–cell fusion activity may be mainly regulated by CDV H/F and SLAM protein at multiple levels, including protein surface expression density, the efficiency of receptor-binding, or the H–F interaction (25–27).

We compared the differences of the amino acid sequence between the rSLAM and mSLAM, and founded difference in residues 60, 74, and 129, ultimately selected amino acids at position 74 and 129 located in the interface of the host–virus interaction as the target sites through SLAM-V homologous modeling method. Furthermore, to study the effect of the two sites on the formation of syncytia, we constructed SLAM mutation expression plasmids and performed surface expression analysis, as well as cell fusion assays. We found that residue 74 had a significant effect on the cell fusion efficiency.

## Materials and Methods

### Cells, Viruses, and Plasmids

BHK-21 (Baby Hamster Kidney, ATCC® CCL-10™) cells were maintained in Dulbecco's modified Eagle's medium (DMEM) with 5% fetal bovine serum (FBS), all cells were cultured at 37°C in the presence of 5% CO_2_. The wild-type CDV strain LN(10)1 (Genbank accession no.KP765764) was isolated from an Arctic fox in 2010 in China. The virus was propagated on Vero cells stably expressing the SLAM receptor from dog (VerodogSLAMtag). Eukaryotic expression plasmids, the pDisplay carrying an HA tag and expressing the SLAM of mink or raccoon dog (pDisplay-mSLAM and pDisplay-rSLAM, respectively), and pCI-H and pCI-F expressing the H/F gene of the CDV LN(10)1 strain were constructed by our laboratory (22). VerodogSLAMtag were obtained from Dr. Veronika von Messling, Paul-Ehrlich-Institute, Germany, and were maintained in DMEM containing 5% FBS (23).

### CDV Infected Cells Stably Expressing rSLAM and mSLAM Receptors

BHK-21 cells (10^5^ cells/mL) were plated in sterile six-well plates and transfected with a SLAM plasmid (pDisplay-mSLAM and pDisplay-rSLAM) when the cell density reached 70–90%. The non-transfected cells were used as the negative control. After 24 h, the medium was replaced with 10% FBS DMEM containing G418 (800 μg/mL) for screening. The medium was replaced every 3 days until more than 99% of the negative control cells died. After two rounds of subculturing, monoclonal cells were selected by the limited dilution method. Then, BHK-rSLAM and BHK-mSLAM were plated in 24-well-plates and infected with an LN(10)1 strain at a multiplicity of infection (MOI) of 0.01. Virus was adsorbed for 1 h at 37°C in serum-free DMEM, and the inoculum was removed and replaced with DMEM containing 10% FBS. Individual wells were collected daily for 6 days, and cell-associated progeny viruses were harvested. Virus titers were determined in VerodogSLAMtag cells by a limiting dilution method and expressed as TCID_50_ using the method of Reed and Muench (28). The assays were repeated twice to determine the mean value.

### Indirect Immunofluorescence Assay (IFA)

BHK-rSALM/ BHK-mSALM cells grown in six-well plates were transfected with pCI-H, and pCI-F plasmid using X-tremeGENE HP DNA Transfection (Roche) according to the manufacturer's instructions. After a 48 h incubation, BHK-rSALM/ BHK-mSALM cells were washed twice with cold PBS, fixed for 20 min with 4% paraformaldehyde, and permeabilized for 30 min with 0.1% Triton X-100. The fixed cells were incubated with anti-CDV H (1:100), for 2 h at 37°C; washed five times with PBS; and then incubated with a fluorescein isothiocyanate (FITC)-labeled goat anti- Mouse IgG (1:100) antibody for 1 h at 37°C. After washing four times with PBS, the cells were examined under a Leica confocal microscope (Leica DMI3000, Germany) with a video documentation system.

### Bioinformatics and Modeling

Multiple sequence alignment was performed by Clustal W (https://www.ebi.ac.uk/Tools/msa/clustalo/) and ESPript 3.0 (http://espript.ibcp.fr/ESPript/ESPript/). Sequence information for CDV LN(10)1-H protein (GenBank: AJP31610.1) and SLAM proteins (dog: AAK61857.1; raccoon dog: ACD47120.1; fox: ACD47119.1; and mink: ACM90097.1) was obtained from the NCBI database. Models of mSLAM: CDV LN(10)1-H and rSLAM: CDV LN(10)1-H complexes were generated using the online tool SWISS-MODEL (https:// swissmodel.expasy.org/interactive#structure) and the MeV-H and maSLAM structures from PDB 3ALZ as templates (29). Three-dimensional (3D) models were constructed and visualized using PyMOL0.99rc2 (Schrödinger LLC, Delano, CA, USA) (30). The interaction between ligands of the protein complex was predicted using the online website PDBePISA (http://www.ebi.ac.uk/msd-srv/prot_int/pistart.html).

### Construction of SLAM Mutants

Following the identification of the two single-nucleotide polymorphisms (SNPs) differentiating the *SLAM* gene, we designed four pairs of primers (Supplementary Table 1) for site-directed mutagenesis using the QuikChange site-directed mutagenesis kit (Agilent, CA, USA) according to the manufacturer's instructions (31). This system was used to construct four single-mutation expression plasmids (pDisplay-rSLAM-I74V, rSLAM-Q129R, mSLAM-V74I, and mSLAM-R129Q) and two double-mutant expression plasmids (pDisplay-rSLAM-I74V/Q129R, and mSLAM-V74I/R129Q).

### Cell Surface Expression of SLAM Protein

At ~70% confluence in a six-well plate, BHK-21 cells were transfected with 4 μg of plasmid per well using 12 μL X-tremeGENE HP DNA Transfection Reagent (Roche, Basel, Switzerland) according to the manufacturer's protocols. After 48 h, cells were digested with 0.25% trypsin, and collected cells were washed with PBS. Cells were suspended in 200 μL 5% bovine serum albumin (BSA) and incubated at room temperature for 30 min. The cells were then washed and incubated sequentially with mouse anti-HA monoclonal antibody (1:50 dilution) at room temperature for 1 h and fluorescein isothiocyanate (FITC)-conjugated goat anti-mouse IgG (1:500 dilution) at room temperature for 30 min. Cells were washed and resuspended in PBS with 1% BSA. The samples were examined by flow cytometry (BD FACSCalibur, USA). Non-transfected BHK-21 cells served as negative controls, combined with FSC and SSC to set up the gates for analysis. Experiments were repeated at least three times.

### Syncytium Formation Assay

BHK-21 cells (10^5^ cells/well) were seeded in six-well plates and co-transfected with three different protein expression vector combinations: (i) pCI-H, (ii) pCI-F, and (iii) each pDisplay-SLAM expressing different SLAMs, using 1.3 μg DNA from each protein expression vector per well. Transfections were performed using 12 μL of X-tremeGENE HP DNA Transfection Reagent (Roche). Eight independent transfection experiments were performed. At 48 h after transfection, cells were fixed, and nuclei were visualized with Hoechst 33342. Images were captured using epifluorescence microscopy (10X magnification). For each transfection experiment, five images were captured, and the average number of syncytia was calculated. In parallel, BHK-21 cells were transfected with SLAM expression plasmids (wild type or derived mutants), and 12 h later, they were infected with LN(10)1 strain at a MOI of 1. After 48–60 h, the average number of syncytia was calculated, and the virus titer was measured as described in CDV Infected Cells Stably Expressing rSLAM and mSLAM Receptors section.

### Statistical Analysis

Statistical analyses were performed with SPSS 16.0 package (SPSS Inc., Chicago, USA), including analysis of variance (ANOVA) and the least significant difference (LSD) *post-hoc* test. *P* < 0.05 was considered to indicate statistical significance. Data analysis results are presented using Prism® 6 for Windows® (GraphPad software, Inc., CA, USA).

## Results

### BHK-rSLAM and BHK-mSLAM Have Different Susceptibilities to LN(10)1

**Figure 1:**
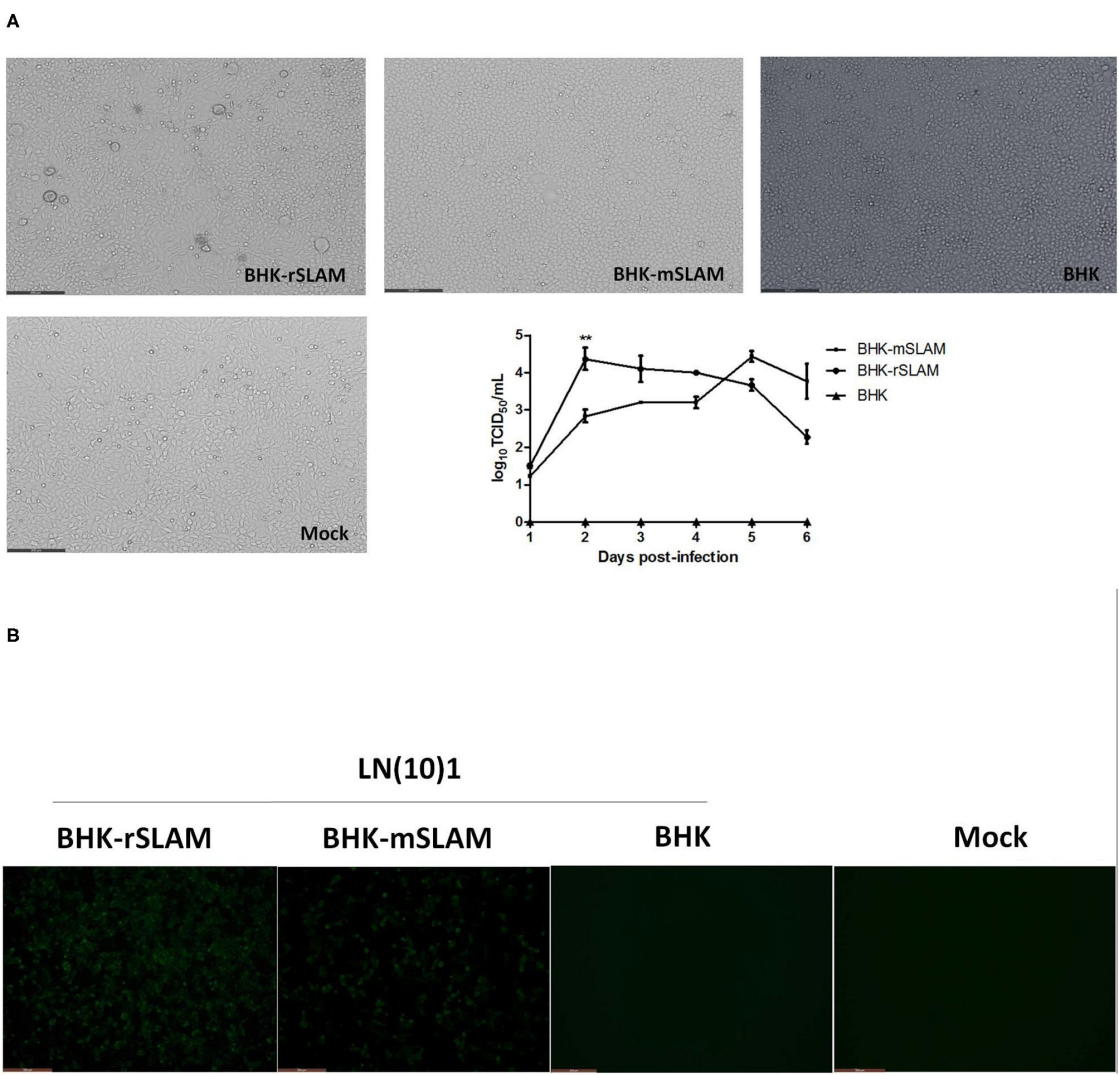
Syncytial formation and virus growth curve. **(A)** Syncytial formation on BHK-rSLAM, BHK-mSLAM, and BHK after CDV LN(10)1 inoculation for 48 h, and virus growth curves of CDV LN(10)1 in BHK-rSLAM and BHK-mSLAM cells. **(B)** Fluorescent images of BHK-rSLAM, BHK-mSLAM, and BHK after CDV LN(10)1 inoculation for 48 h. Mock, untreated BHK cells.

LN(10)1 was used to infect BHK-rSLAM and BHK-mSLAM cells, and the resulting cytopathic effects was observed 48 h later. There were variations in the size of the syncytia caused by the same CDV in cell lines expressing rSLAM and mSLAM. The syncytia occurring in BHK-rSLAM cells were found to be larger and more numerous by direct virus infection (Figure 1A) or IFA (Figure 1B). The virus growth curve also showed that the viral titer in rSLAM cells was significantly higher than that in mSLAM cells on the 2nd day of infection (^**^P < 0.01, Figure 1A). These results correspond to the findings of Zhao et al. (22). and revealed the different *in vitro* susceptibilities of cells expressing rSLAM and mSLAM to the same CDV.

### Mink Has Two SLAM Mutation Sites (I74V and Q129R)

**Figure 2:**
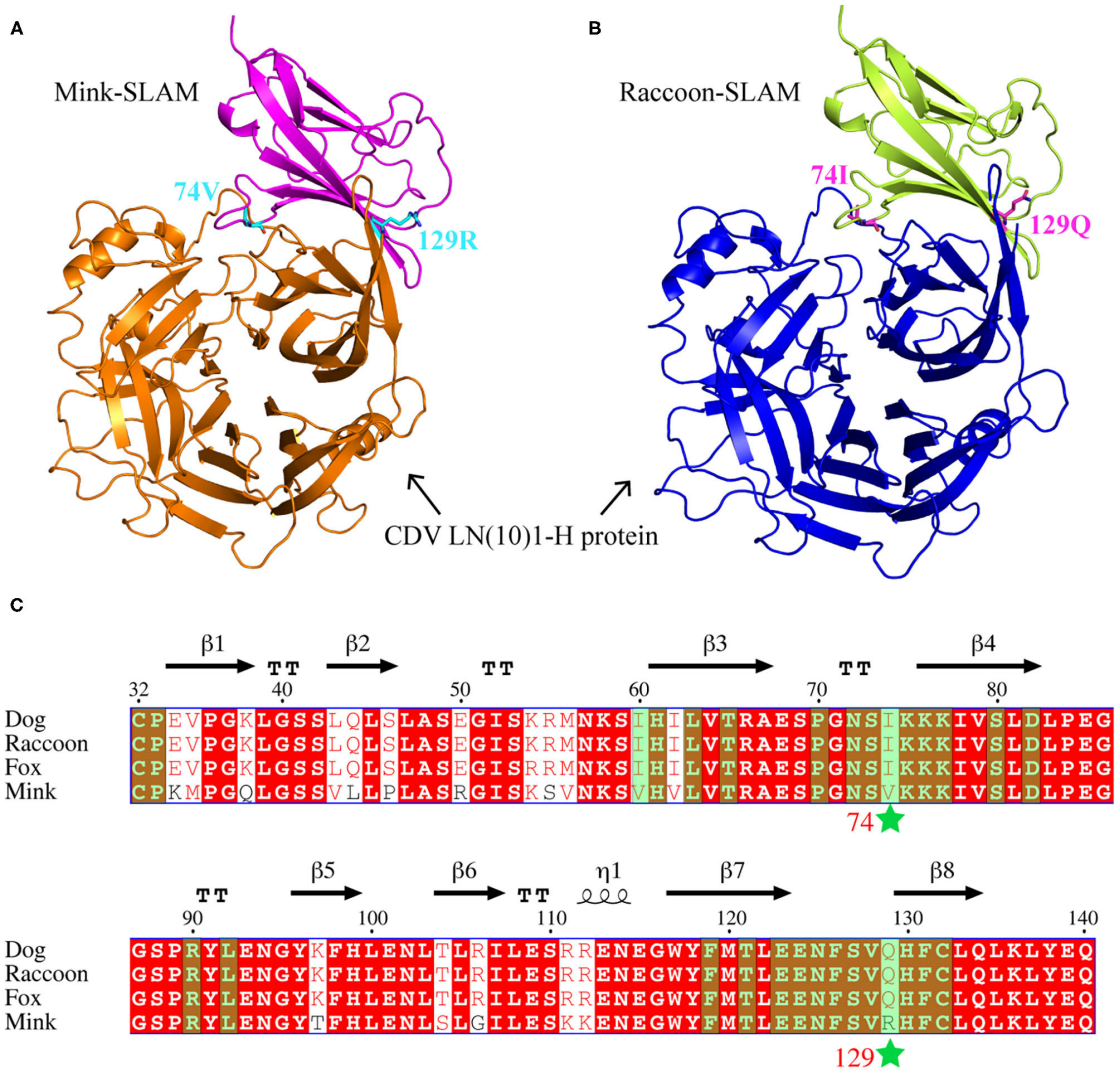
Amino acid sequence alignment and complex models. **(A,B)** Complex models of mSLAM:CDV LN(10)1-H and rSLAM: CDV LN(10)1-H, respectively. **(C)** Structure-based amino acid sequence alignment of the SLAM-V region of four species (dog, fox, raccoon dog, and mink), with the secondary structure elements indicated. Black arrows above the alignment indicate β-strands; cylinders denote α-helices. Two mutation sites (I74V and Q129R) are marked with stars, and the sites that may interact with CDV H are highlighted in green shading.

The SLAM V-CDV H 3D models of mink and raccoon dog were obtained using SWISS-MODEL. The front sheet contained four anti-parallel β-strands and appeared to provide an interface for CDV (Figures 2A,B). Based on our report (22), the amino acid sequences of the SLAM variable (V) regions of cloned dogs, foxes, raccoon dogs, and minks were aligned, and 29 amino acids that could interact with CDV H were predicted by PDBePISA; these are marked in green shading in Figure 2C. We found that the amino acids at these sites are highly conserved. Only residues 60, 74, and 129 were different between canines and minks. The residue 60 is I in canines or V in minks and they are aliphatic. These amino acids 74 and 129 were located at the SLAM interface. R129 of mSLAM was positively charged, but Q129 of rSLAM had no charge, implying a difference in charge between the mink and raccoon SLAM at position 129. These results suggested that residues 74 and 129 may play an important role in the interaction between SLAM and CDV H.

### The mSLAM-V74I Have Significant Effect on the Expression of mSLAM

**Figure 3:**
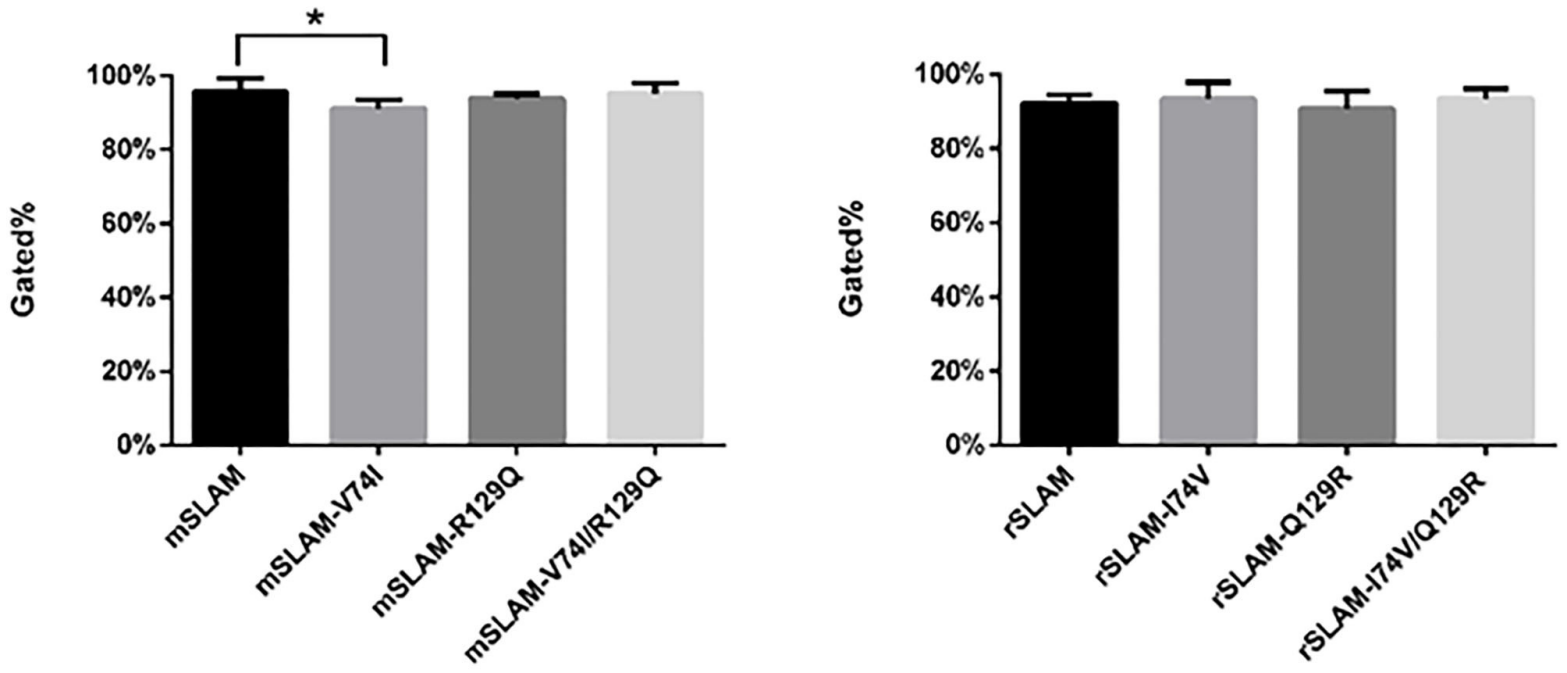
Statistical analysis of flow cytometry results; data represent mean ± standard error. The gated % on the vertical axis indicates the percentage of cells with fluorescence intensity stronger than 10^1^. *P < 0.05.

BHK-21 cells transfected with eight different plasmids (wild type or derived mutants) were subjected to flow cytometry, which showed the expression of SLAM at ~90%. Based on the analysis of four repeated experiments, it was observed that only substitution of residue 74 (mSLAM-V74I) resulted in a significant reduction in mSLAM expression, with a decrease in the average protein expression rate from 95.6% to 91.1% (*P* < 0.05). There were no significant differences in rSLAM expression in cells transfected with rSLAM plasmid with amino acid substitutions at positions 74 and 129 (rSLAM-I74V, rSLAM-Q129R, and rSLAM-I74V/Q129R) (Figure 3).

### Effect of SLAM Mutation on Syncytium Formation Induced by CDV H/F

**Figure 4:**
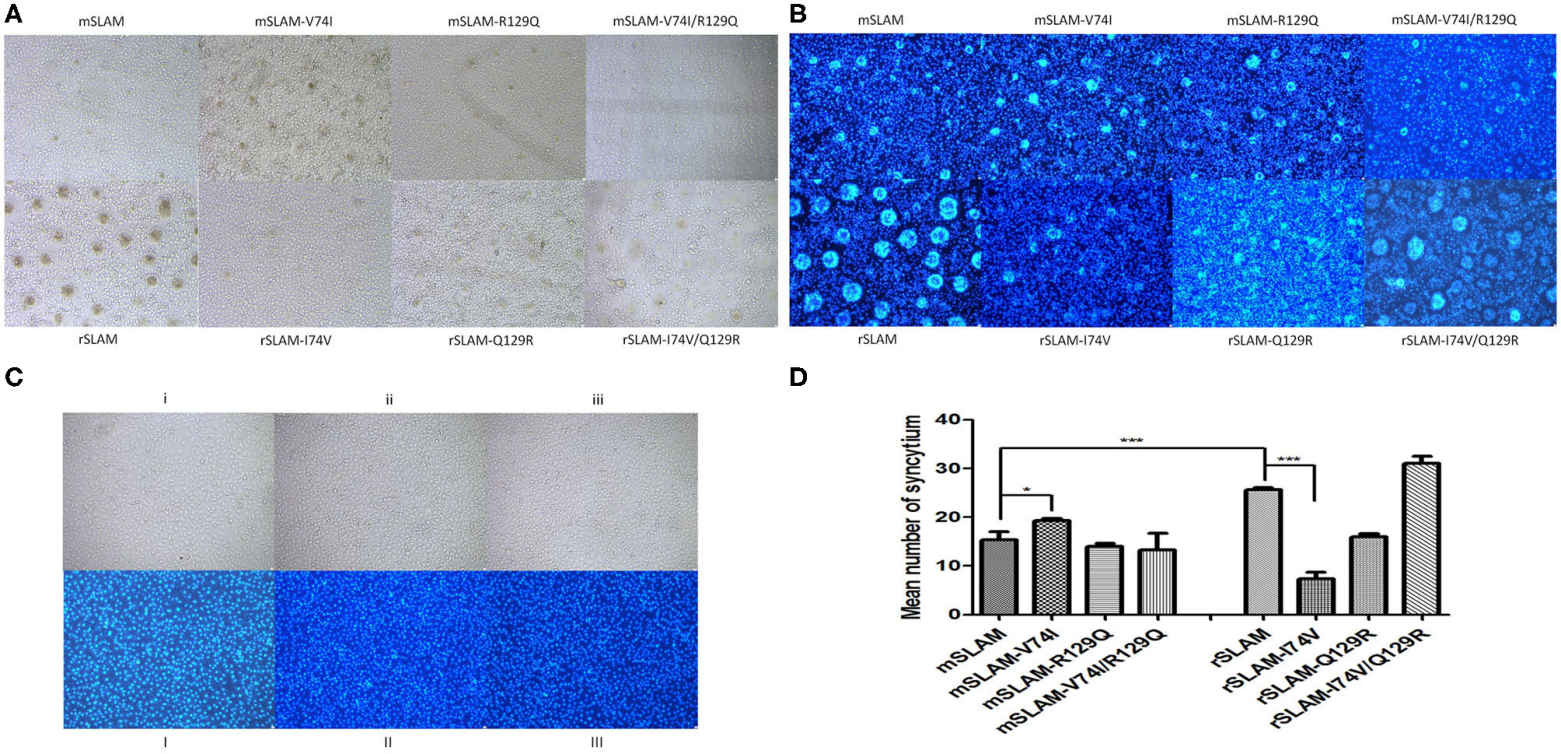
Cell fusion and nuclear staining analysis. **(A)** Cell fusion after co-transfection for 48 h. **(B)** Syncytial staining by Hoechst33342. **(C)** i and I: Transfected with pDisplay-SLAM; ii and II: transfected with pCI-H or pCI-F alone; iii and III: transfected with empty plasmid control (pDisplay and pCI) **(D)** Statistical analysis of mean syncytium number; data represent mean ± standard error. *P < 0.05, ***P < 0.001.

Cell fusion occurred at 24 h after the co-transfection of BHK-21 cells (Figure 4A). After 48 h, the cells were stained with Hoechst33342, and the nuclei were visualized by bright blue fluorescence under a fluorescence microscope. Obvious syncytium formation was observed as clusters of blue fluorescence. It was observed that amino acid substitutions in mink SLAM (mSLAM-V74I) led to an obvious increase in syncytia size and number, while the corresponding amino acid substitutions in raccoon dog SLAM (rSLAM-I74V) had highly significant effects on syncytium size and number (Figure 4B). No fusion was observed in cells transfected with pDisplay-mSLAM, pCI-H, and pCI-F alone and those transfected with empty plasmid combinations (pDisplay and pCI) (Figure 4C).

Amino acid substitution in mSLAM resulted in an increase in the mean number of syncytia (mSLAM: 15.3; mSLAM-V74I: 19.3; mSLAM-R129Q: 14; and mSLAM-V74I/R129Q: 13.3). Of these, the substitution in amino acid 74 alone showed a significant increase in the mean number of syncytia (*P* < 0.05). The rSLAM-I74V/Q129R amino acid substitutions resulted in an increase in the mean number of syncytia (rSLAM, 25.6; rSLAM-I74V, 7.3; rSLAM-Q129R, 16; and rSLAM-I74V/Q129R, 31), while the rSLAM- I74V amino acid substitution significantly decreased the mean number of syncytia (*P* < 0.001, Figure 4D). These results were consistent with the change in the number of syncytia observed visually and confirmed that substitutions in these amino acids had an influence on syncytium formation.

### CDV Infection Analysis

**Figure 5:**
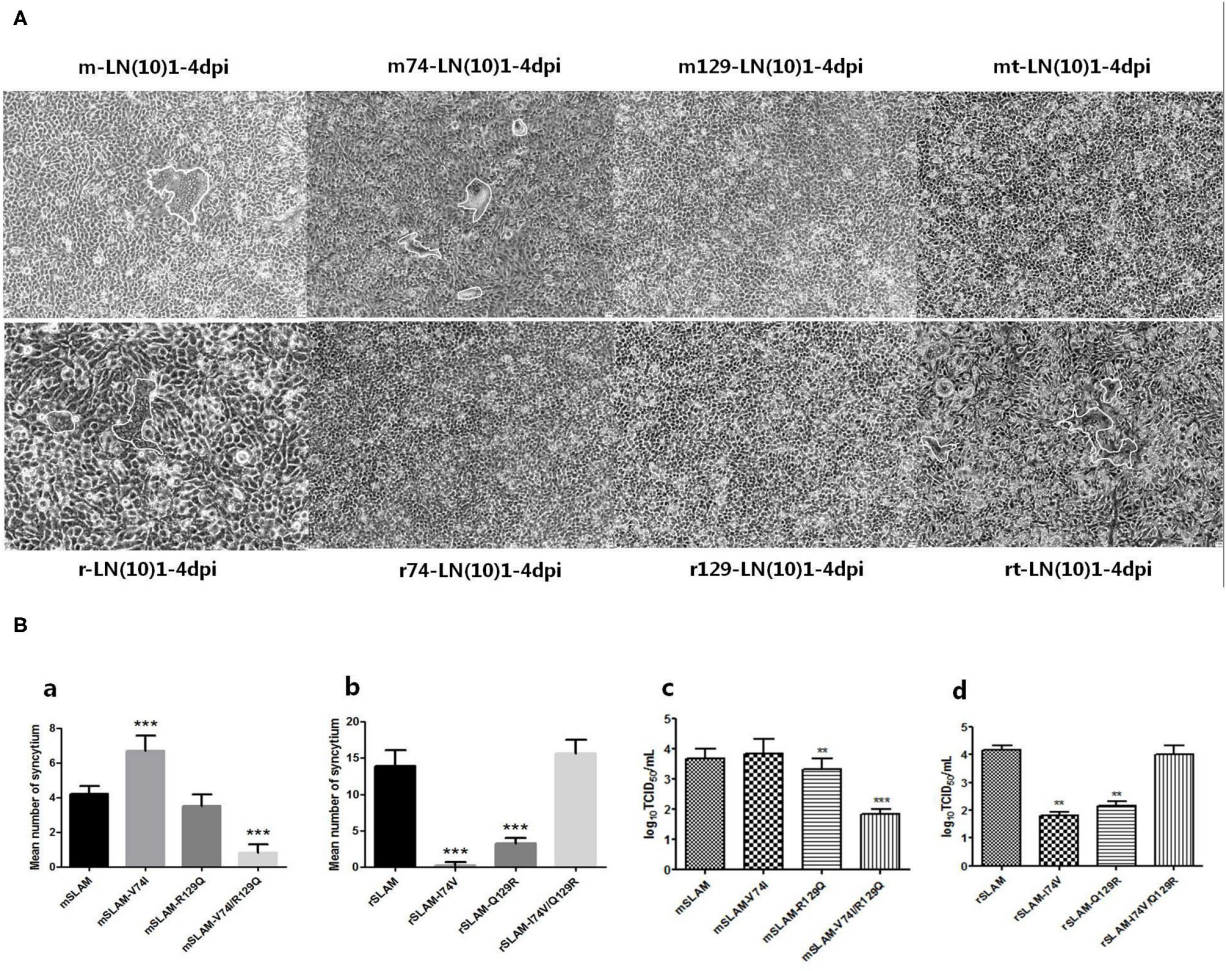
**(A)** Cell fusion after CDV infection for 60 h. **(B)** Syncytial formation and virus titers produced by different SLAM-expressing cells infected with LN(10)1. (a,b) Statistical analysis of mean syncytium number. (c,d) Statistical analysis of virus titers. rt, rSLAM-I74V/Q129R; mt, mSLAM-V74I/R129Q. **P < 0.01, ***P < 0.001.

We used CDV LN(10)1 strains to infect SLAM-expressing cells (wild-type or derived mutants), and the mean number of syncytia significantly varied between different SLAM-expressing cells (Figure 5A, mSLAM, 4.2; mSLAM-V74I, 6.7; mSLAM-R129Q, 3.5; mSLAM-V74I/R129Q, 0.8 and rSLAM, 13.9; rSLAM-I74V, 0.3; rSLAM-Q129R, 3.3; and rSLAM-I74V/Q129R, 15.7). Statistical analysis demonstrated that mSLAM-V74I showed a significant increase in the mean number of syncytia (Figure 5B, *P* < 0.001) and mSLAM-V74I/R129Q significantly decreased the mean number of syncytia (*P* < 0.001), while rSLAM-I74V and rSLAM-Q129R significantly decreased the mean number of syncytia (*P* < 0.001). Consistent with the change in the mean number of syncytia, virus titers at 60 h post infection significantly varied between different SLAM-expressing cells. The LN(10)1 strain produced lower virus titers in cell lines expressing the mSLAM-V74I/R129Q receptor than before mutation (*P* < 0.05), and virus titers in rSLAM-I74V and rSLAM-Q129R cell lines also decreased significantly (*P* < 0.001). Unlike the results described in Effect of SLAM Mutation on Syncytium Formation Induced by CDV H/F section above, infecting SLAM-expressing cells with CDV live virus showed that mutations at amino acids 74 and 129 of SLAM might contribute to viral infection.

## Discussion

SLAM/CD150 is as the major morbillivirus receptor responsible for virus pathogenesis and host range expansion (32). There have been different reports showing that the H-binding region might be responsible for the level of susceptibility or resistance of a species to a particular morbillivirus (33). In 2016, Khosravi et al. (25) confirmed that the E123A substitution in canine SLAM had a pronounced impact on fusion promotion. Previous studies of the interaction between CDV and the SLAM receptor have focused mainly on the analysis of the structure of the CDV H protein and the effects of gene mutations (34–36). Nikolin et al. (37) analyzed the size of syncytia and the mean number of nuclei (MNN) generated by a CDV H-Y549H-induced SLAM-expressing cell line and established a method to assess the efficiency of virus-induced cell fusion. Here, we chose to count the mean number of syncytia because of the small changes in syncytia size and the difficulties associated with determining the MNN. In contrast to previous studies focusing on CDV H proteins, we started the present study by evaluating SLAM receptors from different animals (mink and raccoon dog) to reveal the effect of different SLAMs on CDV-induced cell fusion efficiency. Our findings provide the basis for further studies of the molecular mechanism underlying CDV susceptibilities in different hosts and cross-host transmission.

Our results indicated that CDV-LN(10)1 differed in proliferation and syncytium formation between BHK-rSLAM and BHK-mSLAM cells, which confirmed the results of our previous CDV infection experiments *in vitro* (22), showing the susceptibility of raccoon dogs to CDV. We speculate that this susceptibility was caused by substitutions in key amino acids in the SLAM-V region. Sequence analysis showed that rSLAM and mSLAM differed at residues 60, 74, and 129 among the 29 possible sites involved in H protein interaction, and Shimizu et al. (20) also implied that residues 74 and 129 of SLAM were the key sites for viral action. Therefore, from our findings, we inferred that differences in raccoon dog and mink SLAM-V at amino acids 74 and 129 might affect the functional conformation of SLAM and H protein, changing the affinity between them and causing further alterations in the efficiency of CDV-induced cell membrane fusion. We constructed mutants of raccoon dog and mink SLAM and conducted a series of experiments to verify the effects. The result suggested that only mSLAM-V74I had obvious impact on SLAM expression.

The results of CDV H/F protein transfection into SLAM-expressing cells and CDV LN(10)1-infected SLAM-expressing cells showed that the mean number of syncytia increased significantly with the mSLAM-V74I mutation, while the mean number of syncytia decreased with the rSLAM-I74V mutation. In this regard, we believe that the changes in amino acids 74 and 129 of SLAM might alter the direction of the amino acid side chain along with the change in charge, which affects its interaction and affinity with CDV H and leads to changes in cell fusion efficiency. Interestingly, it was also shown that double mutations in SLAM at positions 74 and 129 seemed to offset the influences caused by single-point amino acid mutations. For example, mSLAM-V74I caused a significant increase in the mean syncytium number, while the number was restored and even lower than mutation-free levels by the mSLAM-V74I/R129Q double mutant. The same was true for rSLAM-I74V/Q129R. We speculated that the simultaneous mutation of amino acids 74 and 129 broke the balance of the hydrogen-bonding network of CDV H and SLAM present in the single-point mutation, resulting in this interesting phenomenon.

Our findings highlight the key role of residue 74 in SLAM, which is located within the interface region of SLAM and is involved in the formation of syncytia induced by CDV. Membrane fusion activity in CDV infections appeared to be finely tuned by a range of subtle molecular interactions between cell-specific receptors and viral glycoproteins. Our results findings provide insights for understanding the differences interaction between CDV and SLAM and laid the foundation for further investigations of the molecular mechanism of CDV transmission and susceptibilities of different hosts.

## Data Availability Statement

The original contributions presented in the study are included in the article/Supplementary Materials, further inquiries can be directed to the corresponding author/s.

## Author Contributions

JZ and XB conceived the study. YW, JC, CG, BH, NS, ML, XB, and JZ were involved in all other aspects of the study, data collection, data analysis, and drafting and editing the paper. All authors have read and agreed to the published version of the manuscript.

## Conflict of Interest

The authors declare that the research was conducted in the absence of any commercial or financial relationships that could be construed as a potential conflict of interest.

## Supplementary Material

Supplementary Table 1 Primer sequences incorporating mutations of amino acids 74 and 129 in mink and raccoon dog SLAM genes.
