# Supplementary material for: Mink SLAM V-Region V74I Substitutions Contribute to the Formation of Syncytia Induced by Canine Distemper Virus
Source: Front Vet Sci. 2021 Jan 21;7:570283. doi: 10.3389/fvets.2020.570283 (PMC7874165; doi:10.3389/fvets.2020.570283)
Supplement: Supplementary Table 1 — Primer sequences incorporating mutations of amino acids 74 and 129 in mink and raccoon dog SLAM genes. [file Table_1.docx]

**Table S1.** Primer sequences incorporating mutations of amino acids 74 and 129 in mink and raccoon dog *SLAM* genes.

| Primer name | Primer sequences (5'-3') |
| --- | --- |
| mSLAM-V74I-F | 5′-tattttcttcttgatactgtttcctggtgattctgctctggt-3′ |
| mSLAM-V74I-R | 5′-accagagcagaatcaccaggaaacagtatcaagaagaaaata-3′ |
| mSLAM-R129Q-F | 5′-cagctgcagacaaaaatgttgaactgaaaagttctcctc-3′ |
| mSLAM-R129Q-R | 5′-gaggagaacttttcagttcaacatttttgtctgcagctg-3′ |
| rSLAM-I74V-F | 5′-gggcggaatcaccgggaaacagtgtcaagaagaaaa-3′ |
| rSLAM-I74V-R | 5′-ttttcttcttgacactgtttcccggtgattccgccc-3′ |
| rSLAM-Q129R-F | 5′-gaggagaacttttcagttcgacacttttgtctgcagctg-3′ |
| rSLAM-Q129R-R | 5′-cagctgcagacaaaagtgtcgaactgaaaagttctcctc-3′ |

*The underlined sites represent the nucleotide sequences used for the mutation
